# Mortality and Morbidity of Urban Road Traffic Crashes in Africa: Capture-Recapture Estimates in Bamako, Mali, 2012

**DOI:** 10.1371/journal.pone.0149070

**Published:** 2016-02-12

**Authors:** Hammadoum A. Sango, Jean Testa, Nicolas Meda, Benjamin Contrand, Mamadou S. Traoré, Pascal Staccini, Emmanuel Lagarde

**Affiliations:** 1 Département d’Enseignement et de Recherche Santé Publique et Spécialités, Faculté de Médecine et d’Odonto-Stomatologie, Université des Sciences, des Techniques et des Technologies de Bamako, Bamako, Mali; 2 Département de Santé Publique. Centre MURAZ, Ministère de la Santé, Bobo-Dioulasso, Burkina Faso; 3 Équipe Prévention et prise en charge des traumatismes. Inserm U897 ISPED—Université de Bordeaux2, Bordeaux, France; 4 Département « Ingénierie du Risque et Informatique de Santé », UMR 912 SESSTIM INSERM, Université de Nice Sophia Antipolis, Nice, France; 5 Centre de Recherche Internationale pour la santé, UFR Sciences de la santé, Université de Ouagadougou, Ouagadougou, Burkina Faso; 6 Institut National de Recherche en Santé Publique (INRSP), Ministère de la Santé et de l’Hygiène Publique, Bamako, Mali; Beihang University, CHINA

## Abstract

**Background:**

Low- and middle-income countries are currently facing the massive public health challenge of road traffic injuries. The lack of effective surveillance systems hinders proper assessment of epidemiologic status and intervention priorities. The objective of our study was to estimate the mortality and morbidity attributable to road crashes in Bamako, Mali using the capture-recapture method.

**Methods:**

During the 1 January, 2012–31 April, 2012 period, we collected data on road traffic crashes from the road accident registers of the police forces of Bamako, Mali on the one hand, and from a register kept by health facilities in the same area. An automatic, then manual matching procedure was performed to find pairs of records related to the same crash victims. The number of victims and the number of fatalities were estimated by the capture-recapture method using the Chapman estimator.

**Results:**

The health facility and the police registries included 3587 and 1432 records, respectively. The matching procedure identified 603 common records, 31 of which were fatalities. The annual incidence estimate for road victims was 1038 in 100 000 and the annual incidence estimate for road fatalities was 12 in 100 000. Victims from both sources were more likely to be male, in the 15–34 age group, and almost half of all injured road users and two in three fatalities were using motorized two-wheelers. One victim out of five was a pedestrian.

**Conclusion:**

Our estimates are in line with available literature data from low-income countries. While more cases were reported by health facilities than by police forces, we believe that an effective surveillance system should not be based solely on medical reports as much would be missing as regards the crash circumstances and characteristics.

## Introduction

According to the last WHO report on road safety, road traffic crashes account for more than 1.24 million deaths and 20 to 50 million injuries each year worldwide. Of all fatalities on the world's roads, 91% occur in low- and middle-income countries, even though these countries have approximately half the world's vehicles [1, 2]. In those countries, more than half of the victims are vulnerable road users (pedestrians, motorcyclists and cyclists) and collective transportation users [3]. Road traffic injuries cause considerable economic losses to victims, their families, and to nations as a whole. Without action, road traffic crashes are predicted to result in the deaths of around 1.9 million people annually by 2020 [4].

A reliable and systematic surveillance system is necessary to monitor road injuries and to target and evaluate relevant intervention programs. In Mali, as in many other African countries [5, 6], such a system is lacking and national statistics rely on standardized forms, filled in by the police, that are either no longer completed or not transmitted [7], resulting in a very incomplete picture of the phenomena.

Even when such a system is operational (as in France), the resulting database underestimates the number of road victims, and in particular vulnerable road users [8, 9]. Capture-recapture analysis using two independent sources of information on the same crash events has been used in countries with a lack of accurate epidemiologic data [5, 10].

We collected available data from hospital and health care centers and from police reports during a 4-month period in Bamako, Mali. Using the capture-recapture method, we estimated the mortality and morbidity attributable to road crashes and compared the relevance of a potential surveillance system based on each data source.

## Materials and Methods

### Data collection

A pilot survey was conducted among all the 15 police stations of the district of Bamako (police forces data source) and was used to build a list of the health facilities receiving the people injured in road traffic accidents where law enforcement officers intervened (health facility data source). This list included Gabriel Touré Hospital, Hospital of Mali, reference health centers of municipalities I, IV, V and VI and the Sébénincoro and Boulkassoumbougou community health centers in municipalities I and IV, respectively. Private health facilities were not included as they only receive victims transferred from other emergency departments.

Reports corresponding to the 1 January, 2012–31 April, 2012 period were selected for the analysis. All 15 police stations of the city of Bamako were provided with a carbon-copy registry in order to collect a copy of all recorded crash reports during the study period. Reports were then coded using a standardized form with variables related to the crash circumstances, to all vehicles and to all individuals involved in a crash in which at least one person was injured, irrespective of the injury severity. All potentially identifying data were collected for matching purposes. For every crash victim attending one of the selected health facilities, specific data were coded using a standardized form with variables related to the crash circumstances, vehicle, and injury location and severity. All potentially identifying data were collected for matching purposes.

### Data matching

The matching procedure was designed to find records from both data sources corresponding to the same individual and to the same crash event. The procedure consisted of two steps. In a first step an automatic probabilistic linkage was computed attributing scores corresponding to the following weighted variables: crash date, crash time, age, sex, last name, first name, place of discharge, crash district, type of road user. All records that the automatic procedure deemed unmatched were tentatively matched manually.

### Capture-recapture analysis

The number of victims and the number of fatalities were estimated by the capture-recapture method using the Chapman estimator [11] as it is less affected by low sample size.

The estimates are given by:

$$\hat{\text{N}}=\frac{{(\text{N}}_{1}{+1)(\text{N}}_{2}+1)}{n+1}-1$$

N_1_ and N_2_ are the number of victims (or fatalities) registered in each respective source n is the number of victims (or fatalities) identified in both sources using the matching procedure.

The variance of these estimates used to provide 95% confidence intervals is given by:

$$\text{V}\hat{\text{ar}}\hat{\left(\text{N}\right)}=\frac{\left({\text{N}}_{1}+\;1)\;({\text{N}}_{2}+\;1)\;({\text{N}}_{1}\;-\;\text{n})({\text{N}}_{2}\;-\;\text{n}\right)}{\left(n+1\right)\left(n+1\right)(n+2)}$$

A stratified capture-recapture analysis was performed to assess the impact of potential differential reporting probabilities according to injury severity, gender, age, vehicle involved, type of road user (driver, passenger or pedestrian), place of discharge.

### Ethics

The protocol was approved by the Institutional Ethics Committee of the Faculty of Medicine and Dentistry; University of Science, Techniques and Technologies of Bamako; Mali.

To participate in this study, participants (patients or guardians) gave their verbal consent.

The study was explained to the patient (if conscious) or guardians in the presence of a member of the emergency room staff in each of the study sites. Only participants who consented were enrolled.

Written consent was not obtained due to the nature of the study, taking place in an ER and the low literacy rate of the general population it was agreed with ethical committee to limit the consenting process to verbal but it was mentioned in the individual file.

Participant consent was recorded by checking "Yes" on the standardized form.

The ethics committees of the Faculty of Medicine and Odonto-Stomatology approved this consent procedure.

Data collection was approved in writing by the Ministry of Health and Hygiene and by the Ministry of Security and Civil Protection of Mali.

## Results

The health facility and police registries included 3587 and 1432 records, respectively. The automatic matching procedure identified 400 victims recorded in both sources and a further 203 victims were manually matched. This led to 603 matched victims (Table 1) 31 of whom were fatalities (Table 2).

**Table 1:** Capture-Recapture Stratified Estimates of Injuries.

	Health facilities		Police			Est. number of events (95% CI)
Stratified by	No. of records	(%)	No. of records	(%)	No. of matches	
**Total**	3587	100%	1432	100%	603	8512 (8041–8982)
**Injuries**						
Nonserious injuries	3085	86%	1198	84%	490	7535 (7066–8004)
Serious injuries	333	11%	77	9%	56	456 (400–512)
Killed	54	2%	57	4%	31	99 (84–113)
Missing	115	3%	100	7%	26	433(312–553)
Total	3587	100%	1432	100%	603	8523(8052–8993)
**Gender**						
Male	2652	74%	1118	78%	457	6481 (6066–6895)
Female	932	26%	314	22%	146	1998 (1782–2214)
Missing	3	0%	0	0%	0	3
Total	3587	100%	1432	100%	603	8482(8012–8953)
**Age in years**						
0–14	439	12%	157	11%	55	1240 (999–1482)
15–24	1255	35%	509	36%	207	3079 (2785–3372)
25–34	937	26%	386	27%	141	2555(2248–2862)
35–44	462	13%	168	12%	73	1056 (892–1221)
45–54	221	6%	75	5%	33	495 (383–608)
55–64	141	4%	54	4%	25	299 (225–374)
65 and over	73	2%	23	2%	11	147 (95–199)
Missing	59	2%	60	4%	58	61 (52–70)
Total	3587	100%	1432	100%	603	8933(8463–9404)
**Vehicle involved**						
Car	170	5%	75	5%	24	519 (368–670)
Small van	19	1%	5	0%	3	29 (15–43)
Truck	14	0%	4	0%	1	37 (6–67)
Bus or coach	123	3%	29	2%	7	464 (212–716)
Motorized two-wheeler	2369	66%	927	65%	403	5443 (5080–5806)
Bike	54	2%	26	2%	7	185 (91–279)
Pedestrian	704	20%	313	22%	124	1770 (1552–1988)
Other	65	2%	35	2%	17	131 (95–167)
Missing	69	2%	18	1%	17	73 (66–79)
Total	3587	100%	1432	100%	603	8650 (8179–9120)
**Road user involved**						
Driver	1937	54%	909	63%	351	5009 (4639–5379)
Passenger	725	20%	223	16%	96	1676 (1443–1908)
Pedestrian	71	2%	169	12%	31	382 (294–469)
Missing	854	24%	131	9%	125	895 (864–925)
Total	3587	100%	1432	100%	603	7961 (7490–8431)
**Place of first discharge**						
Gabriel Touré Hospital	2853	80%	933	65%	463	5744 (5405–6083)
Hospital of Mali	509	14%	144	10%	22	3214 (2061–4367)
CsREF	95	3%	93	6%	11	751 (394–1108))
CsCOM	19	1%	17	1%	0	359 (0–832)
Missing	111	3%	245	17%	107	254 (247–261)
**Total**	3587	100%	1432	100%	603	10 322(9852–10 793)

**Table 2:** Capture-Recapture Stratified Estimates of Deaths.

	Health facilities		Police			Est. number of events (95% CI)
Stratified by	No. of records	(%)	No. of records	(%)	No. of matches	
**Total**	54	100%	57	100%	31	99 (84–113)
**Gender**						
Male	46	85%	45	79%	24	85 (70–101)
Female	8	15%	12	21%	7	14 (12–16)
Missing	0	0%	0	0%	0	0
Total	54	100%	57	100%	31	99 (84–113)
**Age in years**						
0–14	10	19%	14	25%	5	27 (16–37)
15–24	12	22%	12	21%	10	14 (13–16)
25–34	12	22%	11	19%	3	38 (15–61)
35–44	10	19%	7	12%	5	14 (10–17)
45–54	3	6%	5	9%	3	5
55–64	2	4%	4	7%	2	4
65 and over	5	9%	4	7%	3	7 (5–8)
Missing	0	0%	0	0%	0	0
Total	54	100%	57	100%	31	108 (93–123)
**Vehicle involved**						
Car	7	13%	9	16%	7	9
Small van	1	2%	2	4%	1	2
Truck	0	0%	1	2%	0	1
Bus or coach	2	4%	3	5%	0	11 (0–23)
Motorized two-wheeler	26	48%	24	42%	15	41 (34–49)
Bike	3	6%	4	7%	1	9 (3–15)
Pedestrian	12	22%	13	23%	6	25 (16–34)
Other	1	2%	1	2%	1	1
Missing	2	0%	0	0%	0	0
Total	54	96%	57	100%	31	101 (86–116)
**Road user involved**						
Driver	24	44%	22	39%	13	40 (31–49)
Passenger	11	20%	14	25%	10	15 (14–17)
Pedestrian	1	2%	14	25%	1	14
Missing	18	33%	7	12%	7	18
Total	54	100%	57	100%	31	87 (73–102)
Place of first discharge						
Gabriel Touré Hospital	37	69%	52	91%	28	68 (60–77)
Hospital of Mali	17	31%	5	9%	3	26 (14–38)
Missing	0	0%	0	0%	0	0
**Total**	54	100%	57	100%	31	94 (80–109)

Victims from both sources were more likely to be male, in the 15–34 age group, and almost half of all injured road users and two in three fatalities were using motorized two-wheelers. One victim out of five was a pedestrian.

Hospital Gabriel Touré accounted for 80% of all health facility records. 65% of the victims from the police registry were reported to have been sent to this hospital. Reference health centers and community health centers provided only 3% of health facility records and were reported as first discharge facilities in 8% of police reports, with no fatality.

As regards reports on victims (Table 1), more drivers were reported by police forces (63%) than by health facilities (54%) and this information was missing in 9% of police reports as compared with 24% in reports from health facilities. The other important difference between the two sources was related to place of discharge as the information was missing for 17% of all police reports and, as expected, by only 3% in the health facility registry. The number of road victims as estimated by the Chapman method was 8512 (95% confidence interval = [8 041–8 982]). Stratified estimates ranged from 7963 to 10 322 victims. The police sources captured only 17% of these victims and health facilities 42%.

As regards fatalities (Table 2), the total number of deaths estimated by the Chapman method was 99 (95% CI = [84–113]), 58% and 54% being reported by the police and health facilities, respectively. Fewer drivers were reported by the police (39% versus 44%) and the Hospital Gabriel Touré was reported as the place of first discharge for 69% of fatalities according to heath facilities and 91% according to the police. Overall, stratified estimates for fatalities ranged from 87 to 110 road deaths.

The population of Bamako was 2459 million in 2012, leading to an annual incidence estimate for road victims of 1 038 in 100 000 (ranging from 971 to 1259) and an annual incidence estimate for road fatalities of 12 in 100 000 (ranging from 11 to 13).

## Discussion

Road traffic victims as estimated by a capture-recapture procedure were 4 times as numerous as those reported by health facilities and 2 times as numerous as those reported by the police. The factor was about 2 for road deaths when comparing capture-recapture estimates with estimates from either health facility or police records. The number of road deaths per 100 000 population as estimated from the police records was similar to that reported for the country as a whole in 2010 (4.5 deaths per 100 000) as compiled in the WHO country profile provided along with the WHO 2013 road safety report [1]. Our capture-recapture method led to a rate 2 times higher (12 in 100 000), confirming that police files are unable to provide an accurate assessment of road fatality levels [10, 12–14] This observation is typical in low-income countries, but not in high-income countries where police files are often close to comprehensiveness for road deaths [8].

The capture-recapture method has been already used to estimate the number of road victims in several low- and middle-income countries. A similar procedure for estimation of fatalities due to road traffic crashes in 2008 in Karachi, Pakistan indicated an incidence of deaths of 10.6 per 100 000 population [15]. A previous study conducted at the same location 12 years earlier indicated a value of 11.2 road deaths per 100 000 [16]. In Thai Nguyen City in Vietnam [17], the incidence of road traffic injury crashes was estimated by a capture-recapture method (based on the police registry and hospital data) as 1055 per 100 000 population, which is close to our 1038 estimate in Bamako. In Leon Municipality, Nicaragua, Tercero and Andersson estimated in 1993 a much higher annual fatal crash rate of 35.5 per 100 000 with a very different crash configuration pattern [18] when compared with Bamako: motorcyclists represented 11% of all road deaths in Nicaragua and 41% in Bamako, while pedestrians and pedal cyclists accounted for 60% of all road deaths in Nicaragua and 9% in Bamako

Zavareh et al. used the same method to estimate the incidence rate of road deaths as 34 per 100 000, in the province of West Azarbaijan in Iran [19]. At the district level, similar work in Malawi in the Lilongwe district indicated 20 deaths per 100 000 [5].

In summary, estimates from our study in Bamako were very consistent with other study findings from low- or middle-income cities, and lower than those estimated in areas including interurban roads. The WHO estimate for 2010 in Mali was 23.1 per 100 000 [1], computed from the result of a negative binary regression model using a set of predictors (vehicle per capita, road density, gross domestic product…). The difference from our estimate is probably explained by the fact that our study was performed in an urban area.

Another seemingly invariable fact is that the number of road injuries and the number of deaths are greatly underestimated by police force registries, even though they are the main source of road crash statistics in the world.

The main methodological limitations of the capture-recapture method are that it requires an equal likelihood of capture between the two data sources. The stratification according to several road user and crash characteristics shows that this was not the case. The way victims are handled by the police is clearly likely to have effects on the choice of the discharge facility, leading to a positive dependency between the sources, and therefore to an underestimated number of events. Comparing, however, stratified and rough estimates suggests that this dependency led to a limited bias. Another methodological concern is the possibility that some cases captured by one source had a null probability of being captured by another. This can typically emerge on the edge of the study area where the competence areas of both police and health facilities could be open to interpretation.

The accuracy of the matching procedure is of importance in the validity of the capture-recapture method. False matches lead to underestimation of the final mortality and morbidity rates, while missing matches leads to overestimation. We used a large range of variables to minimize false matches. Note that values of those variables were rarely missing, except for the type of road users missing in one record out of four in the health facility victim database and for the place of discharge missing in 17% of police records. To ensure a small number of missing matches, the automatic matching procedure parameters were set so as to produce a large number of “unsure” pairs to be confirmed manually. In manual matching we mainly resorted to the available transcriptions of the names of people involved in the crashes.

The main striking features of road traffic crashes in Bamako include a very large share of deaths of males (85%), aged less than 35 (80%), and a large proportion of deaths of motorized two-wheelers (41%) and pedestrians (25%).

While more cases were reported by health facilities than by police forces, we believe that an effective surveillance system should not be based solely on medical reports. Typically, these data are accurate regarding injury pattern, but much is missing as regards crash circumstances and characteristics. This is illustrated in our data where we observed that data on the type of victim (driver, passenger, pedestrian) is missing in 24% of health facility records as compared with 9% for police registry data. Yet crash setting and environment, road users and vehicles involved and all identifiable risk behaviors are factors that are needed to design appropriate road safety policies.

## References

[1] WHO. Global status report on road safety:supporting a decade of action Genève: WHO; 2013 p. 318.

[2] WHO. The road safety in the world. Report of situation 2009 Genève: WHO; 2009.

[3] Constant A, Lagarde E. Protecting vulnerable road users from injury. PLoS Med. 2010;7: e1000228 10.1371/journal.pmed.1000228 20361017 PMC2846852

[4] WHO. Road accidents: fact sheet n°358. Geneva: World Health Organisation, 2013; 1: 13. Available: http://www.who.int/mediacentre/factsheets/fs358/en/content/1/1/13.

[5] Samuel JC, Sankhulani E, Qureshi JS, Baloyi P, Thupi C, Lee CN, et al Under-reporting of road traffic mortality in developing countries: application of a capture-recapture statistical model to refine mortality estimates. PLoS One 2012;7: e31091 10.1371/journal.pone.0031091 22355338 PMC3280223

[6] Lagarde E. Road traffic injury is an escalating burden in Africa and deserves proportionate research efforts. PLoS Med 2007;4: e170 17593893 10.1371/journal.pmed.0040170 PMC1896192

[7] Republic of Mali. Interministerial order N°3073 / MICT-MSPC-MATCL-SG, carrying institution of the bulletin of analysis of personal accidents (BAAC) in Republic of Mali. In: Presidency of the Republic, editor. Bamako; 2001. p. 6.

[8] Amoros E, Martin JL, Laumon B. Under-reporting of road crash casualties in France. Accid Anal Prev 2006;38: 627–35. 16545764 10.1016/j.aap.2005.11.006

[9] Amoros E, Martin JL, Lafont S, Laumon B. Actual incidences of road casualties, and their injury severity, modelled from police and hospital data, France. Eur J Public Health 2008;18: 360–5. 10.1093/eurpub/ckn018 18381295

[10] Abegaz T, Berhane Y, Worku A, Assrat A, Assefa A. Road traffic deaths and injuries are under-reported in Ethiopia: a capture-recapture method. PLoS One 2014;9: e103001 10.1371/journal.pone.0103001 25054440 PMC4108419

[11] Chapman DG, University of California B. Some properties of the hypergeometric distribution with applications to zoological sample censuses. Berkeley: University of California Press; 1951.

[12] Dhillon PK, Lightstone AS, Peek-Asa C, Kraus JF. Assessment of hospital and police ascertainment of automobile versus childhood pedestrian and bicyclist collisions. Accid Anal Prev 2001;33: 529–37. 11426683 10.1016/s0001-4575(00)00066-x

[13] Kudryavtsev AV, Nilssen O, Lund J, Grjibovski AM, Ytterstad B. Road traffic crashes with fatal and non-fatal injuries in Arkhangelsk, Russia in 2005–2010. Int J Inj Contr Saf Promot 2013;20: 349–57. 10.1080/17457300.2012.745576 23216194

[14] Tercero F. Injury occurrence in developing countries Can valid and prevention-oriented information on injury occurrence be obtained from existing data sources in developing countries? An example from Nicaragua. Int J Consum Product Safety Promotion 1998; 5: 99–105.

[15] Lateef MU. Estimation of fatalities due to road traffic crashes in Karachi, Pakistan, using capture-recapture method. Asia Pac J Public Health 2010;22: 332–41. 10.1177/1010539509356808 21212049

[16] Razzak JA, Luby SP. Estimating deaths and injuries due to road traffic accidents in Karachi, Pakistan, through the capture-recapture method. Int J Epidemiol 1998;27: 866–70. 9839745 10.1093/ije/27.5.866

[17] Van HT, Singhasivanon P, Kaewkungwal J, Suriyawongpaisal P, Khai LH. Estimation of non-fatal road traffic injuries in Thai Nguyen, Vietnam using capture-recapture method. Southeast Asian J Trop Med Public Health 2006;37: 405–11. 17125007

[18] Tercero F, Andersson R. Measuring transport injuries in a developing country: an application of the capture-recapture method. Accid Anal Prev 2004;36: 13–20. 14572822 10.1016/s0001-4575(02)00109-4

[19] Khorasani Zavareh D, Mohammadi R, Laflamme L, Naghavi M, Zarei A, Haglund BJ. Estimating road traffic mortality more accurately: use of the capture-recapture method in the West Azarbaijan province of Iran. Int J Inj Contr Saf Promot 2008;15: 9–17. 10.1080/17457300701794105 18344091

